# Supplementary material for: Medication-Wide Association Study Plus (MWAS+): A Proof of Concept Study on Drug Repurposing
Source: Med Sci (Basel). 2022 Aug 31;10(3):48. doi: 10.3390/medsci10030048 (PMC9503040; doi:10.3390/medsci10030048)
Supplement: Supplementary file 1 [file medsci-10-00048-s001.zip › medsci-1820240-supplementary.pdf]

Supplementary Materials: Alzheimer's Disease and Alzheimer's Disease-Related Dementias in Older African American and White Veterans.

Table S1. AD/ADRD Diagnosis Codes

**ICD9/10 Code**	**Code Description**
**ICD9 Code**	
290.0	Senile dementia, uncomplicated
290.10	Presenile dementia, uncomplicated
290.4*	Vascular dementia
291.2	Alcohol-induced persisting dementia
292.82	Drug-induced persisting dementia
294.1*	Dementia in conditions classified elsewhere
294.2*	Dementia, unspecified
294.8	Non-specific dementia
331.0	Alzheimer's disease
331.19	Other frontotemporal dementia
331.2	Senile degeneration of brain
331.7	Cerebral degeneration in diseases classified elsewhere
331.82	Dementia with Lewy bodies
331.89	Other cerebral degeneration
331.9	Cerebral degeneration, unspecified
**ICD10 Code**	
F01.5*	Vascular dementia
F02*	Dementia in other diseases classified elsewhere
F03*	Unspecified dementia
F10.27	Alcohol dependence with alcohol-induced persisting dementia
F10.97	Alcohol use, unspecified with alcohol-induced persisting dementia
F13.27	Sedative, hypnotic or anxiolytic dependence with sedative, hypnotic or anxiolytic-induced persisting dementia
F13.97	Sedative, hypnotic or anxiolytic use, unspecified with sedative, hypnotic or anxiolytic-induced persisting dementia
F18.17	Inhalant abuse with inhalant-induced dementia
F18.27	Inhalant dependence with inhalant-induced dementia
F18.97	Inhalant use, unspecified with inhalant-induced persisting dementia
F19.17	Other psychoactive substance abuse with psychoactive substance-induced persisting dementia
F19.27	Other psychoactive substance dependence with psychoactive substance-induced persisting dementia
F19.97	Other psychoactive substance use, unspecified with psychoactive substance- induced persisting dementia
G30*	Alzheimer's disease
G31.09	Other frontotemporal dementia
G31.83	Dementia with Lewy bodies

Table S2. Excluded Condition Codes

**ICD9/10 Code**	**Code Description**
**ICD9 Code**	
Schizophrenia	

295*	Schizophrenic disorders
Bipolar Disorder	
296.0*	Bipolar I disorder, single manic episode
296.4*	Bipolar I disorder, most recent episode (or current) manic
296.5*	Bipolar I disorder, most recent episode (or current) depressed
296.6*	Bipolar I disorder, most recent episode (or current) mixed
296.7*	Bipolar I disorder, most recent episode (or current) unspecified
296.8*	Other and unspecified bipolar disorders
Dementia of Known Causes	
046.1*	Jakob-Creutzfeldt disease
046.3	Progressive multifocal leukoencephalopathy
046.79	Other and unspecified prion disease of central nervous system
046.9	Unspecified slow virus infection of central nervous system
331.11	Pick's disease
333.0	Other degenerative diseases of the basal ganglia
333.4	Huntington's chorea
**ICD10 Code**	
Schizophrenia	
F20*	Schizophrenia
Bipolar Disorder	
F31*	Bipolar disorder
Dementia of Known Causes	
A81.00	Creutzfeldt-Jakob disease, unspecified
A81.01	Variant Creutzfeldt-Jakob disease
A81.09	Other Creutzfeldt-Jakob disease
A81.2	Progressive multifocal leukoencephalopathy
A81.89	Other atypical virus infections of central nervous system
F10.96	Alcohol use, unspecified with alcohol-induced persisting amnestic disorder
G23.1	Progressive supranuclear ophthalmoplegia [Steele-Richardson-Olszewski]
G31.01	Pick's disease
G90.3	Multi-system degeneration of the autonomic nervous system
